# Constructing Stable and Potentially High-Performance Hybrid Organic-Inorganic Perovskites with “Unstable” Cations

**DOI:** 10.34133/2020/1986576

**Published:** 2020-06-02

**Authors:** Qing Yang, Menghao Wu, Xiao Cheng Zeng

**Affiliations:** ^1^School of Physics, Huazhong University of Science and Technology, Wuhan, Hubei 430074, China; ^2^Department of Chemistry, University of Nebraska, Lincoln, NE 68588, USA

## Abstract

A new family of functional hybrid organic-inorganic perovskites (HOIPs) is theoretically designed based on the following chemical insights: when a proton is adhered to molecules like water or ethanol, the newly formed larger-sized cations (e.g., H_5_O_2_^+^, C_2_H_5_OH_2_^+^, and CH_3_SH^+^) entail low electron affinities mimicking superalkalis; they are conjugated acids of weak bases that cannot survive in solution, while their chemistry behavior in the HOIP frameworks, however, may be markedly different due to greatly enhanced cohesive energies of the proton, which facilitate the formation of new HOIPs. First-principles computations show that the putative formation reactions for these newly designed HOIPs typically release much more energy compared with the prevailing HOIP MAPbI_3_, suggesting the likelihood of facile solution-based fabrications, while the suppression of reverse formation suggests that the humidity stability may be markedly enhanced. During their formations, halide acids are unlikely to react with ethanol or methanethiol without the presence of metal halides, a condition further favoring their stability. The proposed structure of (H_5_O_2_)PbI_3_ may also clarify the origin of the long-speculated existence of HPbI_3_. Importantly, density functional theory computations suggest that many of these HOIPs possess not only direct bandgaps with values within the optimal range for solar light absorbing but also more desirable optical absorption spectra than that of MAPbI_3_, where their ferroelectric polarizations also benefit photovoltaics. The stability and photovoltaic efficiency may be even further improved for the newly designed two-dimensional (2D) HOIPs and 2D/3D hybrid HOIP structures.

## 1. Introduction

Hybrid organic-inorganic perovskites (HOIPs) have attracted intense attention for their high performance in photovoltaics [1–4], achieving a record of power conversion efficiency higher than 25% within several years [5, 6]. The general formula of most HOIPs can be written as ABX_3_, where A is an organic cation like methylammonium CH_3_NH_3_^+^ (MA^+^) or formamidinium [NH_2_]_2_CH^+^ (FA^+^), B is a metal cation like Pb^2+^ or Sn^2+^, and X is a halide anion [1, 7–11]. Historically, HOIP was first fabricated by replacing Cs cations in bulk CsPbI_3_ with organic groups [12]. Nitrogen-containing organic groups like MA or FA possess low ionization energy (IE), i.e., low electron affinity (EA) of their cations, mimicking superalkalis [13, 14]. Today, fabrication of HOIPs can be greatly facilitated by mixing the AX and BX_2_ precursors in solution or in vapor phase, which may be applicable for industry production. For example, MAPbI_3_ can be synthesized via the reaction PbI_2_ + MAI⟶MAPbI_3_, forming new Pb-I bonds and bulk perovskite structure. However, the insertion process of MAI species into the 2D layered PbI_2_ can be sluggish due to the long diffusion path and strong structure reconstruction, resulting in nonuniform films and incomplete coverage. The synthesis can be further improved by using HPbI_3_ as a new precursor to replace the lead halide [15, 16]. The proposed HPbI_3_ has been a hypothetical structure without any structural or spectroscopic confirmation. Several theoretical studies revealed that HPbI_3_ is not chemically stable and it readily dissociates into PbI_2_ and HI [17, 18]. It is well known that the IE of the H atom is >13 eV, a very high value distinctly different from those of alkali metal or MA, whose IE values are <6 eV. Moreover, it is known that stable perovskite structures often satisfy the “tolerance factor,” defined as

(1) $$\begin{array}{c} t=\frac{r_{\text{A}}+r_{\text{X}}}{\sqrt{2}\left(r_{\text{B}}+r_{\text{X}}\right)}, \end{array}$$

where *r*_A_, *r*_B_, and *r*_X_ represent the effective ionic radii of A, B, and X ions, and *t* should be empirically within the range of 0.76-1.13. The proton radius is obviously too small and is outside the tolerance factor range for making stable perovskite structures with other ions.

Note that once clustering with water molecules occurs, the proton behaves like alkali cations with much reduced EA and the enlarged ion radius. These value-added features may facilitate the formation of stable perovskite structure. Our ab initio calculations show that HPbI_3_ is highly unstable while the reaction PbI_2_ + HI + 2H_2_O⟶(H_5_O_2_)PbI_3_ is energetically favorable. This result may clarify the previously speculated existence of HPbI_3_. The putative formation reaction is akin to the formation of the tin-based compounds like (H_5_O_2_)SnI_3_, which may render fabrication possible by using a mixture of metal dihalides and halide acids as the precursors. Note that this reaction appears to entail much lower formation energy compared with that of the reaction PbI_2_ + MAI⟶MAPbI_3_. Once bound with some other stable molecules (e.g., C_2_H_5_OH, CH_3_SH), protons may also behave like alkali cations with much reduced EA for making stable perovskites due to the low formation energies. Moreover, we show that several newly predicted HOIPs also possess direct bandgaps in the optimal range of 0.9-1.6 eV as solar absorbers. The ferroelectric polarizations of these newly predicted HOIPs also benefit photovoltaics.

## 2. Results

**Figure 1:**
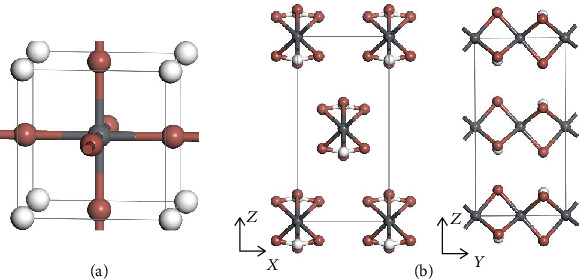
Geometric structure of the (a) cubic and (b) orthorhombic phases of HPbI_3_. Grey, brown, and white spheres denote Pb, I, and H atoms, respectively. Both phases are energetically unfavorable.

First, we examined various possible structures of HPbI_3_. Based on our calculations, the cubic perovskite structure given in the Materials Project database [19] turns out to be highly energetically unfavorable (Figure 1(a)), since a high energy of 3.31 eV/f.u. can be released if decomposed into PbI_2_ and HI. We construct an orthorhombic phase with the CmCm symmetry based on face-sharing [PbI_6_] hexagonal stacking structure. This new structure is 2.80 eV/f.u. lower in energy than the cubic structure. As shown in Figure 1(b), the H atoms are bonded to the surface iodine atoms in the [PbI_3_] chains. Still, such a new structure is still energetically unfavorable since the energy cost Δ*E* for the reaction PbI_2_ + HI⟶HPbI_3_ is 0.51 eV/f.u., which would be endothermic. The unfavorable energetics is due to the small radius of H and high EA of the proton. However, when the protons are attached to water molecules in solution, larger-sized cations with lower EA would be formed.

**Figure 2:**
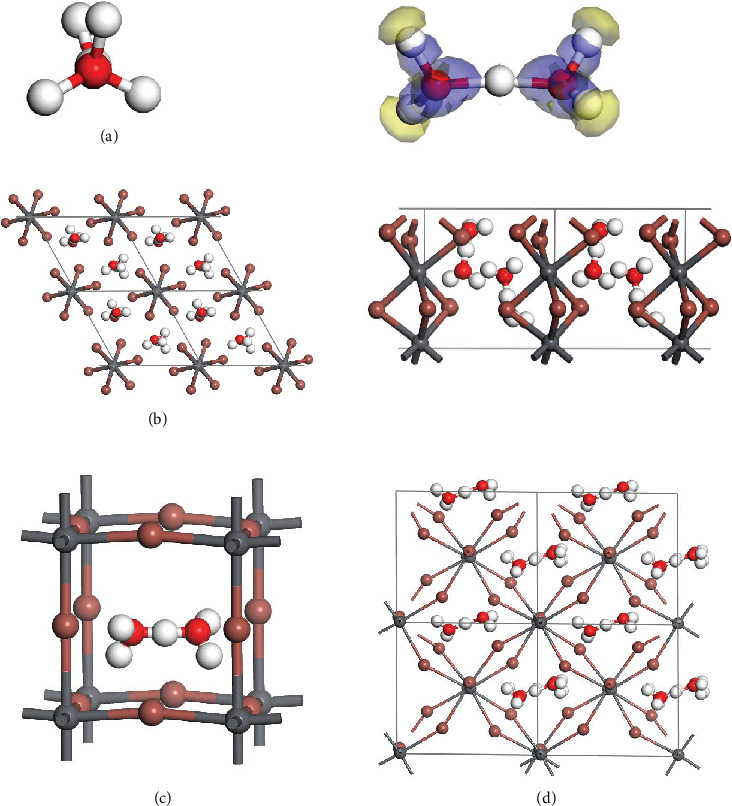
(a) Structure and computed electron deformation density (side view) of cation H_5_O_2_^+^. Structure of the (b) orthorhombic, (c) cubic, and (d) tetragonal phases of (H_5_O_2_)PbI_3_.

Next, we calculate the EA of proton H^+^ binding with 1 and 2 water molecules: H_3_O^+^ and H_5_O_2_^+^, which are, respectively, reduced to 6.4 and 3.1 eV, where the latter can be deemed as a superalkali cation. The ground-state geometry of cation H_5_O_2_^+^ is shown in Figure 2(a), where the proton is located at the center of two water molecules. The distances between the central proton and two O atoms are both 1.21 Å, a value between that of the covalent O-H bond (usually 1.0~1.1 Å) and that of the hydrogen bond (usually 1.4~1.6 Å). The electron of central H in H_5_O_2_^+^ is highly delocalized according to the computed electron deformation density. The noncentrosymmetric structure should give rise to a dipole moment. Apart from lower EA, the radius of the cation is also much enlarged, and such larger-sized cations may lead to stable perovskite structures once combined with PbI_2_. We constructed two perovskite structures by replacing the protons in Figure 1(b) (orthorhombic) and Figure 1(a) (cubic) by H_5_O_2_^+^, respectively, as shown in Figures 2(b) and 2(c). The computed energy change Δ*E* for the formation reaction PbI_2_ + HI + 2H_2_O⟶(H_5_O_2_)PbI_3_ is, respectively, reduced to -0.043 and 0.167 eV/f.u. Here, the lattice parameters (*a* = *b* = 8.64 Å, *c* = 8.13 Å) for the orthorhombic structure shown in Figure 2(b) appear to be in good agreement with the reported values from fitting the X-ray diffraction pattern [16], and the proposed formation reaction is exothermal, which may account for previously claimed existence of HPbI_3_.

Note that for the prevailing perovskite MAPbI_3_, a previous experiment indicates two observed phase transitions, one at 327.4 K from tetragonal to cubic structure and another at 162.2 K from orthorhombic to tetragonal structure [20]. Previous DFT calculations [21, 22] show that the energy cost for the reaction PbI_2_ + MAI⟶MAPbI_3_ is, respectively, 0.1 eV/f.u. and -0.003 eV/f.u. for the formation of the cubic phase and orthorhombic phase. If the cubic structure of (H_5_O_2_)PbI_3_ is replaced by the tetragonal structure in Figure 2(d), which is 0.170 eV/f.u. lower in energy, the reaction would be exothermal with Δ*E* = −0.003 eV/f.u. as well. Meanwhile, thermal stability of the cubic phase of (H_5_O_2_)SnI_3_ at the room temperature can be examined by using ab initio molecular dynamics (AIMD) simulations with the system temperature being controlled at 300 K. The final structure at the end of 5 ps is still intact (see Fig. S1), without showing transformation into the orthorhombic phase. For other alkali atoms, e.g., Li, with much smaller radius compared with Cs, the EA can be reduced from 5.25 to 4.20 eV when attached to a water molecule. As such, the energy cost of the formation of LiPbI_3_ and NaPbI_3_ is, respectively, reduced by 1.33 and 0.47 eV/f.u. upon the intercalation of a water molecule attaching to each alkali cation, as shown in Fig. S2.

The formation of the same group of compounds, such as (H_5_O_2_)SnI_3_ and (H_5_O_2_)PbBr_3_, even the cubic phase, can be more energetically favorable:

(2) $$\begin{array}{c} \text{Sn}{\text{I}}_{2}+\text{HI}+2{\text{H}}_{2}\text{O}⟶\left({\text{H}}_{5}{\text{O}}_{2}\right)\text{Sn}{\text{I}}_{3},\Delta E=-0.03\text{ eV}/\text{f}.\text{u}.\\ \text{PbB}{\text{r}}_{2}+\text{HBr}+2{\text{H}}_{2}\text{O}⟶\left({\text{H}}_{5}{\text{O}}_{2}\right)\text{PbB}{\text{r}}_{3},\Delta E=-0.09\text{ eV}/\text{f}.\text{u}.\\ \text{SnB}{\text{r}}_{2}+\text{HBr}+2{\text{H}}_{2}\text{O}⟶\left({\text{H}}_{5}{\text{O}}_{2}\right)\text{SnB}{\text{r}}_{3},\Delta E=-0.12\text{ eV}/\text{f}.\text{u}. \end{array}$$

So, their solution-based synthesis might be proceeded via deposition of metal dihalide MX_2_ and halide acid HX mixture.

**Figure 3:**
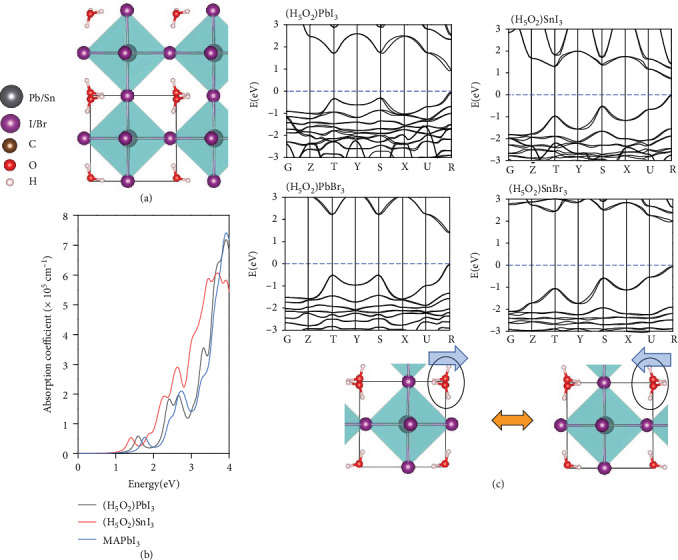
(a) Computed band structures of the cubic phase (H_5_O_2_)PbI_3_, (H_5_O_2_)SnI_3_, (H_5_O_2_)PbBr_3_, and (H_5_O_2_)SnBr_3_. (b) Computed optical absorption spectra of (H_5_O_2_)PbI_3_ and (H_5_O_2_)SnI_3_. (c) Ferroelectric switching of (H_5_O_2_)SnI_3_. The blue arrows denote the polarization direction of cations and the whole crystal.

We calculate the band structures of the newly predicted stable perovskites by using the HSE+SOC method (see Figure 3(a)). These perovskites all possess direct bandgaps of 0.74, 1.43, and 2.02 eV, respectively. (H_5_O_2_)SnI_3_ exhibits perhaps the lowest bandgap among the HOIPs computed using the same approach in the literature. To achieve higher photovoltaic performance (e.g., a Shockley-Queisser efficiency of ~25%), direct bandgaps within the optimal range of 0.9-1.6 eV are desired. Here, (H_5_O_2_)PbBr_3_ seems to be an ideal candidate as it possesses a modest bandgap and low Δ*E*. Note that the photovoltaic performance of most current HOIPs is still poor in the infrared region [26], while the low bandgap HOIPs like (H_5_O_2_)SnI_3_ may fill the gap. The computed optical absorption spectrum (see Figure 3(b)) suggests even stronger absorption compared with MAPbI_3_ [23], rendering (H_5_O_2_)SnI_3_ a promising candidate as an efficient solar absorber. If the compound of a narrow-bandgap ABX_3_ and a wide-bandgap A′BX_3_ can be fabricated as A*_x_*A′_1-*x*_BX_3_, a wider range of bandgaps may be attained via controlling composition *x*, which may be tuned such that the Shockley-Queisser detailed balance limit of photovoltaic conversion efficiency for monobandgap semiconductors could be overpassed. Here, the narrow bandgap of (H_5_O_2_)SnI_3_ and wide bandgap of (H_5_O_2_)SnBr_3_ may be complementary to one another to cover a wider range of spectra. Note also that the absence of centrosymmetry of cation H_5_O_2_^+^ in Figure 2(a) gives rise to polarity. With the reorientations of these polar cations in (H_5_O_2_)SnI_3_ as marked by the circles shown in Figure 3(c), where the blue arrows denote the polarization direction of cations and the whole crystal, a ferroelectric polarization of 22.7 *μ*C/cm^2^ would emerge, much higher than the previously predicted polarization value of MAPbI_3_ [24]. Theoretically, ferroelectricity may benefit photovoltaics because high photovoltage can be induced by polarization, while photogenerated electrons and holes can be separated by a built-in electric field [25, 26]. Moreover, combination of a high-mobility narrow-bandgap semiconductor and nonvolatile memory is also desirable [27, 28].

**Figure 4:**
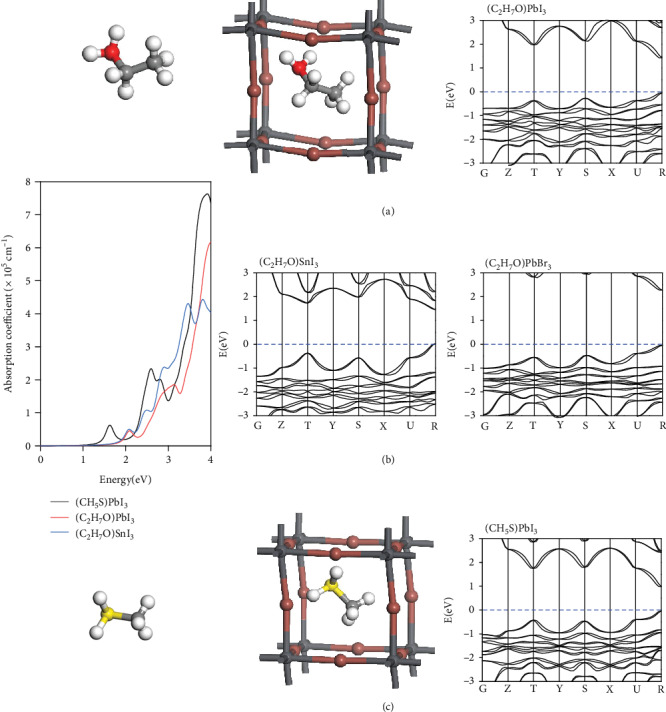
(a) Structure of C_2_H_5_OH_2_^+^ cation and related HOIPs and the band structures of HOIPs. (b) Computed optical absorption spectra of (CH_5_S)PbI_3_, (C_2_H_5_OH_2_)PbI_3_, and (C_2_H_5_OH_2_)SnI_3_. (c) Structures of CH_3_SH_2_^+^ cation and (CH_3_SH_2_)PbI_3_ and the band structures of (CH_3_SH_2_)PbI_3_.

Upon solution mixing of a metal dihalide MX_2_ and halide acid HX, the halide anions may disassociate from the halide acids and join in the skeleton of the framework, while the protons may be attached to the oxygen atoms of water molecules and form a low-EA cation, mimicking MA^+^. Aside from water molecules, the proton may also bind with oxygen-containing organic molecules and become cations that may give rise to new and stable HOIPs. Ethanol, for example, can become a cation with EA of 5.27 eV when binding with a proton at the site of the oxygen atom, as shown in Figure 4(a). As such, energetically favorable perovskite structures can be constructed:

(3) $$\begin{array}{c} \text{Pb}{\text{I}}_{2}+\text{HI}+{\text{C}}_{2}{\text{H}}_{5}\text{OH}⟶\left({\text{C}}_{2}{\text{H}}_{5}\text{O}{\text{H}}_{2}\right)\text{Pb}{\text{I}}_{3},\Delta E=-0.09\text{ eV}/\text{f}.\text{u}.\\ \text{Sn}{\text{I}}_{2}+\text{HI}+{\text{C}}_{2}{\text{H}}_{5}\text{OH}⟶\left({\text{C}}_{2}{\text{H}}_{5}\text{O}{\text{H}}_{2}\right)\text{Sn}{\text{I}}_{3},\Delta E=-0.28\text{ eV}/\text{f}.\text{u}.\\ \text{PbB}{\text{r}}_{2}+\text{HBr}+{\text{C}}_{2}{\text{H}}_{5}\text{OH}⟶\left({\text{C}}_{2}{\text{H}}_{5}\text{O}{\text{H}}_{2}\right)\text{PbB}{\text{r}}_{3},\Delta E=-0.24\text{ eV}/\text{f}.\text{u}. \end{array}$$

These compounds might be formed in a solution of metal dihalides, halide acids, and ethanol. Here, the halide acids are unlikely to react with ethanol without the presence of metal halides, a condition further favoring the stability of the product HOIPs. In contrast, the high stability of MAI actually disfavors and even lowers the stability of the product MAPbI_3_, as it only takes 0.003 eV/f.u. for MAPbI_3_ to decompose into MAI and PbI_2_ as mentioned above. The thermal stability of (C_2_H_5_OH_2_)PbI_3_ is examined via AIMD simulations as shown in Fig. S1. The cubic structure seems be stable at 300 K. Aside from oxygen, the sulfur atom in a molecule can also be the binding site for the proton. For example, methanethiol can become a cation with EA of 5.33 eV when binding with a proton, as shown in Figure 4(c). The formation reaction for the perovskite with such cations is exothermal: PbI_2_ + HI + CH_3_SH⟶(CH_3_SH_2_)PbI_3_, Δ*E* = −0.34 eV/f.u. These perovskite structures are energetically much more favorable than MAPbI_3_, considering their much more energy release upon the formation. Figures 4(a) and 4(c) also display the computed band structures of (C_2_H_5_OH_2_)PbI_3_, (C_2_H_5_OH_2_)SnI_3_, (C_2_H_5_OH_2_)PbBr_3_, and (CH_3_SH_2_)PbI_3_. These perovskites possess direct bandgaps of 1.48, 1.47, 2.31, and 1.03 eV, respectively, all within the optimal range of 0.9-1.6 eV except (C_2_H_5_OH_2_)PbBr_3_. According to the Mulliken charge analysis, the average charges on organic cation, Pb ion, and I ion are, respectively, 0.65 *e*, 0.49 *e*, and −0.38 *e* for (C_2_H_5_OH_2_)PbI_3_ and 0.62 *e*, 0.40 *e*, and −0.34 *e* for (CH_3_SH_2_)PbI_3_. It seems that (C_2_H_5_OH_2_)PbI_3_ is more ionic, which may explain why it possesses a wider bandgap compared with (CH_3_SH_2_)PbI_3_. It might be the similar case for (C_2_H_5_OH_2_)PbBr_3_ with an even wider bandgap as Br is more electronegative than I. The computed optical absorption spectra are shown in Figure 4(b), exhibiting even stronger absorption compared with that of MAPbI_3_. The major factor that causes the difference in the optical absorption might be attributed to different bandgaps, as shown by the redshift of the spectrum for (CH_3_SH_2_)PbI_3_ due to its smaller bandgap compared with other HOIPs. Similarly, the absence of centrosymmetry of cations (as shown in Figures 4(a) and 4(c)) would also give rise to polarity. With the reorientations of these polar cations as marked by the circles shown in Fig. S3(c), where the blue arrows denote the polarization direction of cations and the whole crystal, considerable polarizations would emerge for both (C_2_H_5_OH_2_)PbI_3_ and (CH_4_SH)PbI_3_. Distinct from (H_5_O_2_)SnI_3_, here, their polarizations possess projection along both the −*x* and −*y* axes in Fig. S3. The computed *P*_*x*_ and *P*_*y*_ are, respectively, 11.08 and 10.98 *μ*C/cm^2^ for (C_2_H_5_OH_2_)PbI_3_ and 7.65 and 1.68 *μ*C/cm^2^ for (CH_4_SH)PbI_3_.

**Figure 5:**
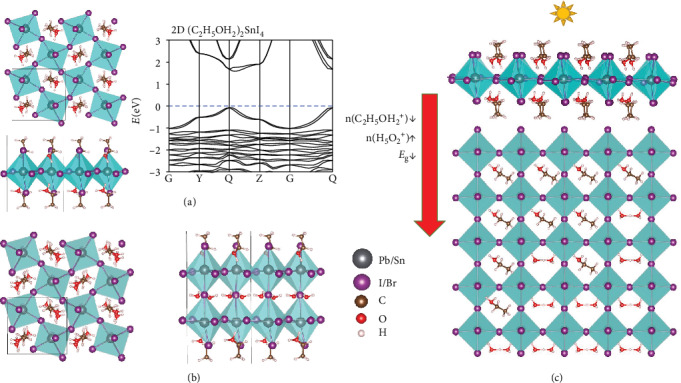
Geometric structure (top and side view) of the 2D (a) (C_2_H_5_OH_2_)_2_SnI_4_ with its band structure and (b) (H_5_O_2_)(C_2_H_5_OH_2_)_2_Pb_2_I_7_. (c) A sketch of design by dripping ethanol on the surface of (H_5_O_2_)SnI_3_ to form perovskite (side and top view). The red arrow denotes the dripping direction.

Depending on the radius of the cations, the 1D, 2D, or 3D perovskite frameworks may be formed: the 3D frameworks tend to form with smaller cations like MA, while larger cations such as Ph(CH_2_)2NH_3_ (PEA) [29, 30] tend to form low-dimensional structures. We note that the cation (C_2_H_5_OH_2_)^+^ is already larger than MA^+^. A 2D structure of (C_2_H_5_OH_2_)_2_SnI_4_ is constructed in Figure 5(a), a typical Ruddlesden-Popper perovskite structure consisting a layer of corner-sharing [SnI_6_] octahedral interleaved with (C_2_H_5_OH_2_)^+^ cations on both surfaces. The following formation reactions of the same group of compounds are all exothermic:

(4) $$\begin{array}{c} \text{Pb}{\text{I}}_{2}+2\text{HI}+2{\text{C}}_{2}{\text{H}}_{5}\text{OH}⟶{\left({\text{C}}_{2}{\text{H}}_{5}\text{O}{\text{H}}_{2}\right)}_{2}\text{Pb}{\text{I}}_{4},\Delta E=-0.57\text{ eV}/\text{f}.\text{u}.\\ \text{Sn}{\text{I}}_{2}+2\text{HI}+2{\text{C}}_{2}{\text{H}}_{5}\text{OH}⟶{\left({\text{C}}_{2}{\text{H}}_{5}\text{O}{\text{H}}_{2}\right)}_{2}\text{Sn}{\text{I}}_{4},\Delta E=-0.65\text{ eV}/\text{f}.\text{u}.\\ \text{Pb}{\text{I}}_{2}+2\text{HBr}+2{\text{C}}_{2}{\text{H}}_{5}\text{OH}⟶{\left({\text{C}}_{2}{\text{H}}_{5}\text{O}{\text{H}}_{2}\right)}_{2}\text{PbB}{\text{r}}_{4},\Delta E=-0.70\text{ eV}/\text{f}.\text{u}. \end{array}$$

Here, the energy release per each organic cation is even higher compared to the formation of the corresponding 3D structures in Figure 4(a); thereby, the low-dimensional structures are more likely to be formed. The thermal stability of (C_2_H_5_OH_2_)_2_PbI_4_ is examined via AIMD simulations (Fig. S1); and the 2D structure can be stable at 350 K. Previous studies of the 2D Ruddlesden-Popper perovskite usually show higher stability but an undesirable wide bandgap (~3 eV) compared with the 3D perovskites. Notably, the bandgap of the 2D (C_2_H_5_OH_2_)_2_SnI_4_ is ~1.7 eV (Figure 5(a)), which is in the desirable range. If water is also involved in the precursors, the smaller cations H_5_O_2_^+^ may be intercalated in the central layer of the 2D Ruddlesden-Popper perovskite structure, as shown in Figure 5(b):

(5) $$\begin{array}{c} 2\text{Pb}{\text{I}}_{2}+3\text{HI}+2{\text{H}}_{2}\text{O}+2{\text{C}}_{2}{\text{H}}_{5}\text{OH}⟶\left({\text{H}}_{5}{\text{O}}_{2}\right){\left({\text{C}}_{2}{\text{H}}_{5}\text{O}{\text{H}}_{2}\right)}_{2}\text{P}{\text{b}}_{2}{\text{I}}_{7},\Delta E=-0.46\text{ eV}/\text{f}.\text{u}.\\ 2\text{Sn}{\text{I}}_{2}+3\text{HI}+2{\text{H}}_{2}\text{O}+2{\text{C}}_{2}{\text{H}}_{5}\text{OH}⟶\left({\text{H}}_{5}{\text{O}}_{2}\right){\left({\text{C}}_{2}{\text{H}}_{5}\text{O}{\text{H}}_{2}\right)}_{2}\text{S}{\text{n}}_{2}{\text{I}}_{7},\Delta E=-0.77\text{ eV}/\text{f}.\text{u}.\\ 2\text{PbB}{\text{r}}_{2}+3\text{HBr}+2{\text{H}}_{2}\text{O}+2{\text{C}}_{2}{\text{H}}_{5}\text{OH}⟶\left({\text{H}}_{5}{\text{O}}_{2}\right){\left({\text{C}}_{2}{\text{H}}_{5}\text{O}{\text{H}}_{2}\right)}_{2}\text{P}{\text{b}}_{2}\text{B}{\text{r}}_{7},\Delta E=-0.77\text{ eV}/\text{f}.\text{u}. \end{array}$$

Finally, we present a structure design, akin to a chemical multijunction, for achieving spatially varying bandgaps over a wide range, which may potentially surpass the Shockley-Queisser detailed balance limit of photovoltaic conversion efficiency for monobandgap semiconductors. Figure 5(c) illustrates a schematic plot of dripping ethanol on the surface of (H_5_O_2_)SnI_3_. With the permeation of ethanol, a density gradient of cations can be formed. The formation energy of (C_2_H_5_OH_2_)SnI_3_ is lower than (H_5_O_2_)SnI_3_, so H_5_O_2_^+^ can be substituted. Since the following reactions are mentioned above,

(6) $$\begin{array}{c} \text{Sn}{\text{I}}_{2}+\text{HI}+2{\text{H}}_{2}\text{O}⟶\left({\text{H}}_{5}{\text{O}}_{2}\right)\text{Sn}{\text{I}}_{3},\Delta E=-0.03\text{ eV}/\text{f}.\text{u}.\\ \text{Sn}{\text{I}}_{2}+\text{HI}+{\text{C}}_{2}{\text{H}}_{5}\text{OH}⟶\left({\text{C}}_{2}{\text{H}}_{5}\text{O}{\text{H}}_{2}\right)\text{Sn}{\text{I}}_{3},\Delta E=-0.28\text{ eV}/\text{f}.\text{u}. \end{array}$$

the reaction below can be inferred:

(7) $$\begin{array}{c} \left({\text{H}}_{5}{\text{O}}_{2}\right)\text{Sn}{\text{I}}_{3}+{\text{C}}_{2}{\text{H}}_{5}\text{OH}⟶\left({\text{C}}_{2}{\text{H}}_{5}\text{O}{\text{H}}_{2}\right)\text{Sn}{\text{I}}_{3}+2{\text{H}}_{2}\text{O},\Delta E=-0.25\text{ eV}/\text{f}.\text{u}. \end{array}$$

The computed bandgap decreases from 1.47 eV of (C_2_H_5_OH_2_)SnI_3_ at the surface to 0.73 eV of (H_5_O_2_)SnI_3_ inside the structure due to the decreasing density as C_2_H_5_OH_2_^+^ substitutes H_5_O_2_^+^ during the permeation of ethanol. On the surface, with the highest density of ethanol, the 2D structure of (C_2_H_5_OH_2_)_2_SnI_4_ may be formed if the HI solution is dripped in the final step.

Likewise, since the following reaction is mentioned above,

(8) $$\begin{array}{c} \text{Sn}{\text{I}}_{2}+2\text{HI}+2{\text{C}}_{2}{\text{H}}_{5}\text{OH}⟶{\left({\text{C}}_{2}{\text{H}}_{5}\text{O}{\text{H}}_{2}\right)}_{2}\text{Sn}{\text{I}}_{4},\Delta E=-0.65\text{ eV}/\text{f}.\text{u}. \end{array}$$

the reactions below can be inferred:

(9) $$\begin{array}{c} \left({\text{H}}_{5}{\text{O}}_{2}\right)\text{Sn}{\text{I}}_{3}+\text{HI}+2{\text{C}}_{2}{\text{H}}_{5}\text{OH}⟶{\left({\text{C}}_{2}{\text{H}}_{5}\text{O}{\text{H}}_{2}\right)}_{2}\text{Sn}{\text{I}}_{4}+2{\text{H}}_{2}\text{O},\Delta E=-0.62\text{ eV}/\text{f}.\text{u}.\\ \left({\text{C}}_{2}{\text{H}}_{5}\text{O}{\text{H}}_{2}\right)\text{Sn}{\text{I}}_{3}+\text{HI}+{\text{C}}_{2}{\text{H}}_{5}\text{OH}⟶{\left({\text{C}}_{2}{\text{H}}_{5}\text{O}{\text{H}}_{2}\right)}_{2}\text{Sn}{\text{I}}_{4},\Delta E=-0.37\text{ eV}/\text{f}.\text{u}. \end{array}$$

Such a 2D layer covered by hydrophobic ligands and with a higher bandgap of 1.7 eV may be utilized as a cover layer to protect the perovskite structure, while enhancing the light absorption in the ultraviolet region. Meanwhile, the switchable polar ligands also give rise to an in-plane 2D ferroelectricity of 1.2 × 10^−10^C/m, which can facilitate the separation of electrons and holes. Such a structure design may render high photovoltaic efficiency due to the desirable spatially varying bandgaps, ranging from 0.7 to 1.7 eV.

## 3. Discussions

It is known that the structure instability of MAPbI_3_ stems in part from the tendency of reversing the formation reaction. As mentioned above, for PbI_2_ + MAI⟶MAPbI_3_, Δ*E* = −3 meV/f.u., so the energy cost of reverse reaction is only 3 meV/f.u. However, for the abovementioned formation reactions such as SnI_2_ + HI + C_2_H_5_OH⟶(C_2_H_5_OH_2_)SnI_3_, Δ*E* = −0.28 eV/f.u.; PbI_2_ + HI + CH_3_SH⟶(CH_3_SH_2_)PbI_3_, Δ*E* = −0.34 eV/f.u.; and SnI_2_ + 2HI + 2C_2_H_5_OH⟶(C_2_H_5_OH_2_)_2_SnI_4_, Δ*E* = −0.65 eV/f.u., the corresponding reverse reactions become very unlikely due to the high energy cost. Their structural stability might be partially attributed their moderate tolerance factors *t*, which are calculated by using equation (1) and listed in Table S1. For stable structures, *t* should be empirically within the range of 0.76-1.13. It turns out that the *t* factors for all the structures with cations H_5_O_2_^+^, C_2_H_5_OH_2_^+^, or CH_3_SH^+^ as listed in Table S1 are within this range. The thermal stabilities of the three compounds are also examined (see Fig. S1).

The chemical stability of the newly designed perovskites may be unexpected, as the cations considered in this study are conjugated acids of very weak bases (H_5_O_2_^+^, pKa = −1.74; C_2_H_5_OH_2_^+^, pKa = −2.4; and CH_3_SH_2_^+^, pKa = −5.4) in the Brønsted–Lowry acid-base theory. Our calculations show that the binding energy for a proton attaching to a C_2_H_5_OH or CH_3_SH molecule is 0.41 or 0.85 eV lower compared to that of H_3_O^+^ (binding a proton to a water molecule). As such, (C_2_H_5_OH_2_)^+^ and (CH_3_SH_2_)^+^ are less likely to form in solution as the protons tend to bind with water molecules. However, the adsorption of the proton would be enhanced in the solid HOIP frameworks. For example, the energy cost for a water molecule to take away a proton from the 2D (C_2_H_5_OH_2_)_2_PbI_4_ would amount to 2.3 eV. It has been suggested in previous studies [31, 32] that the poor humidity stability of MAPbI_3_ can be attributed to the trapped charges:

(10) $$\begin{array}{c} {\text{I}}^{-}+{\text{H}}_{2}\text{O}⟶\text{HI}+\text{O}{\text{H}}^{-}\\ \text{O}{\text{H}}^{-}+{\text{H}}_{2}\text{O}⟶{\text{H}}_{2}\text{O}+\text{O}{\text{H}}^{-}\\ \text{C}{\text{H}}_{3}\text{N}{{\text{H}}_{3}}^{+}+\text{O}{\text{H}}^{-}⟶\text{C}{\text{H}}_{3}\text{N}{\text{H}}_{2}+{\text{H}}_{2}\text{O} \end{array}$$

By similar simulation with a trapped charge, however, the energy cost for a hydroxyl ion to take away a proton from (H_5_O_2_)PbI_3_ and (CH_3_SH_2_)PbI_3_ would be, respectively, 1.94 and 1.80 eV, indicating potentially high humidity stability. Since there are other possible mechanisms of decomposition, we cannot guarantee that the newly designed systems can survive in the high humidity. However, it is highly likely that their humidity stability is markedly enhanced compared with the benchmark perovskite MAPbI_3_.

## 4. Conclusions

In conclusion, we have shown a series of superalkali cations with a relatively large radius and much reduced electron affinity (e.g., H_5_O_2_^+^, C_2_H_5_OH_2_^+^, and CH_3_SH_2_^+^) through adding protons to prevailing molecules like water or ethanol, which notably enrich the number of cation candidates for perovskite synthesis. Even though these cations are less stable in solution due to their chemical nature of conjugated acids of weak bases, they become much more stable within the perovskite frameworks due to their high binding energies with the proton. As a result, they could potentially form new and stable hybrid perovskites like (H_5_O_2_)SnI_3_, (C_2_H_5_OH_2_)SnI_3_, and (CH_3_SH_2_)PbI_3_. For these compounds, the formation reactions are predicted to be exothermic with much more energy release compared with the prevailing MAPbI_3_, which may enable facile solution-processed fabrication, and the reverse reactions will be suppressed due to the high energy costs. Many of these newly predicted perovskites possess not only direct bandgaps within the optimal range for solar absorbing but also more desired optical absorption spectra compared with that of MAPbI_3_. Lastly, the 2D and 2D/3D hybrid perovskite structures are also predicted to be energetically highly favorable due to their high formation energies. If confirmed in the laboratory, special perovskite designs with novel spatially varying bandgaps over a wide energy range may be achieved to offer high photovoltaic efficiency.

## 5. Computational Methods

Our density functional theory (DFT) calculations are performed using the Vienna Ab initio Simulation Package (VASP 5.3.4) code [33, 34]. The electron-ion interaction is described by the projector augmented wave (PAW) method [35], and the generalized gradient approximation (GGA) in the Perdew-Burke-Ernzerhof (PBE) form is applied [36]. The plane wave cutoff energy for a wave function is set to 500 eV. The 9 × 9 × 9 Monkhorst-Pack *k*-point mesh is employed for sampling the Brillouin zone [37]. The lattice parameters and atomic positions are fully relaxed until the energy difference is less than 10^−6^ eV and the force on each atom is smaller than 0.01 eV/Å. Since the conventional GGA functional like PBE tends to underestimate the bandgaps, the Heyd-Scuseria-Ernzerhof (HSE06) hybrid functional is also used to compute the electronic structures and optical properties [38] while taking into account the spin-orbit coupling (SOC) effect. As for the 2D structure, a vacuum region of 15 Å is added to the vertical direction to neglect the interaction between the neighboring layers. According to previous reports [39, 40], the accuracy of DFT computations can be enhanced by using dispersion correction when calculating dispersion effects in perovskites and related compounds. We have examined our results by using the D2 functional of Grimme [41] to take the van der Waals interaction into account. Taking the formation reaction PbI_2_ + HI + 2H_2_O⟶(H_5_O_2_)PbI_3_ as a paradigmatic case, Δ*E* computed by using PBE+D2 is 0.187 eV/f.u., slightly higher than 0.167 eV/f.u. based on the PBE functional.

The Berry phase method is employed to evaluate crystalline polarization [42]. For optical properties, we first compute the frequency-dependent complex dielectric function and then the absorption coefficient based on the equation:

(11) $$\begin{array}{c} \alpha \left(\bar{\omega }\right)=\frac{\sqrt{2}e}{\hbar c}{\left[{\left({\varepsilon_{1}}^{2}+{\varepsilon_{2}}^{2}\right)}^{1/2}-{\varepsilon_{1}}^{2}\right]}^{1/2}, \end{array}$$

where *ε*_1_ and *ε*_2_ are the real and imaginary parts of the dielectric function, respectively. The formation energy associated with the reaction A + B + C⟶D is computed based on the formula, Δ*E* = *E*(D) − *E*(A) − *E*(B) − *E*(C), where each term is the energy of the corresponding crystal structure. The ab initio molecular dynamics (AIMD) simulations are also performed using the VASP code, where the canonical ensemble, the algorithm of Nosè, and a time step of 1 fs are adopted.

## Supplementary Materials

Supplementary Materials Figure S1: snapshots of (H_5_O_2_)PbI_3_ and (C_2_H_5_OH_2_)PbI_3_ at 300 K and (C_2_H_5_OH_2_)_2_PbI_4_ at 350 K, taken from ab initio molecular dynamics simulations at the end of 5 ps. Figure S2: geometric structure of Na(H_2_O)PbI_3_. Table S1: tolerance factors of various cubic HOIPs. Figure S3: ferroelectric switching of (a) (C_2_H_5_OH_2_)PbI_3_ and (b) (CH_4_SH)PbI3. The blue arrows denote the polarization directions.
