# Supplementary material for: Constructing Stable and Potentially High-Performance Hybrid Organic-Inorganic Perovskites with “Unstable” Cations
Source: Research (Wash D C). 2020 Jun 2;2020:1986576. doi: 10.34133/2020/1986576 (PMC7288242; doi:10.34133/2020/1986576)
Supplement: Supplementary Materials — Figure S1: snapshots of (H5O2)PbI3 and (C2H5OH2)PbI3 at 300 K and (C2H5OH2)2PbI4 at 350 K, taken from ab initio molecular dynamics simulations at the end of 5 ps. Figure S2: geometric structure of Na(H2O)PbI3. Table S1: tolerance factors of various cubic HOIPs. Figure S3: ferroelectric switching of (a) (C2H5OH2)PbI3 and (b) (CH4SH)PbI3. The blue arrows denote the polarization directions. [file 1986576.f1.docx]

Supplementary Material

**Constructing Stable and Potentially High-Performance Hybrid Organic-Inorganic Perovskites with “Unstable” Cations**

Qing Yang^1^, Menghao Wu^1,^*, Xiao Cheng Zeng^2,^*

^1^School of Physics, Huazhong University of Science and Technology, Wuhan, Hubei, 430074, China

^2^Department of Chemistry, University of Nebraska, Lincoln, NE, 68588, USA


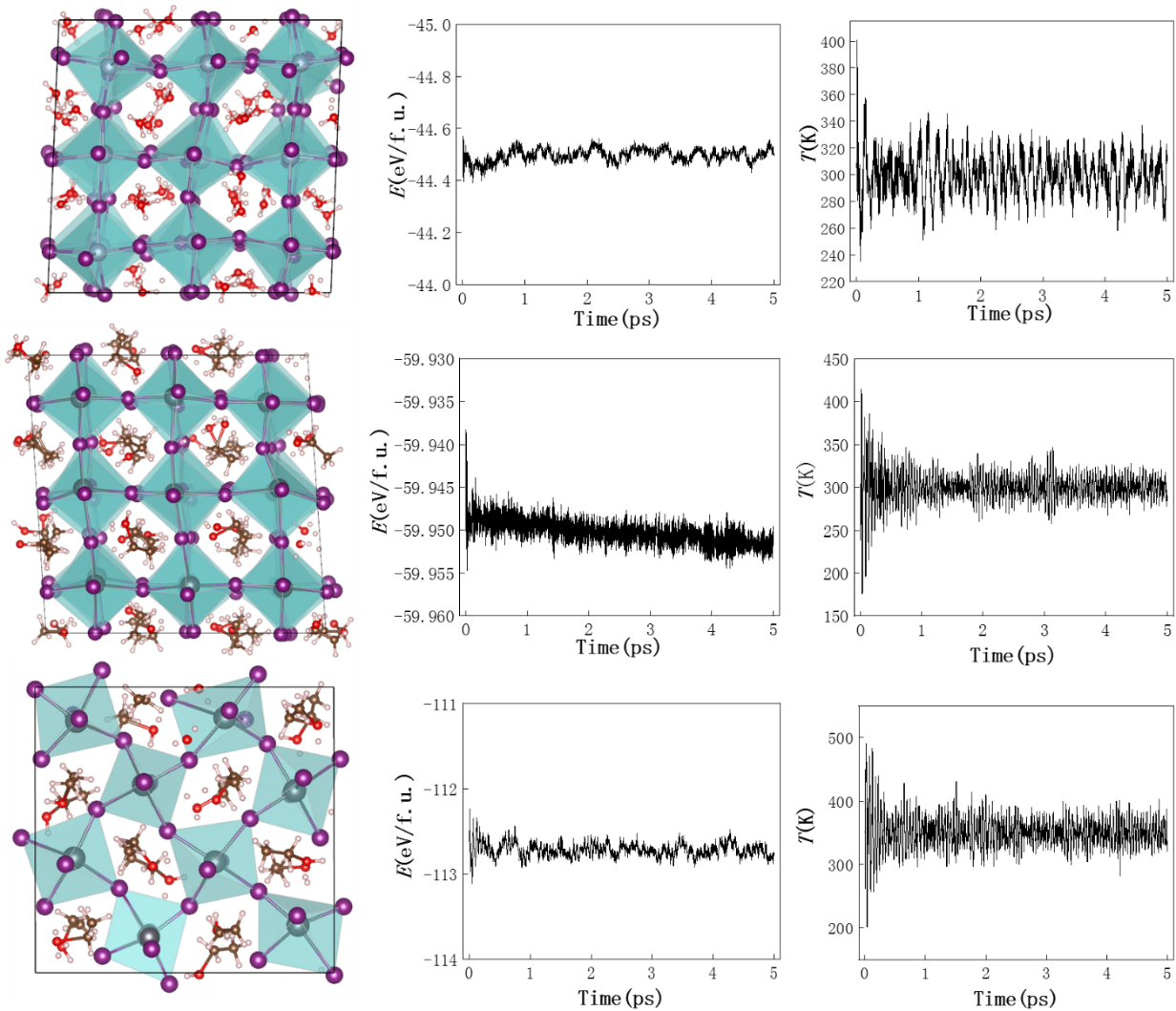


Figure S1. Snapshots of (H_5_O_2_)PbI_3_ and (C_2_H_5_OH_2_)PbI_3_ at 300 K and (C_2_H_5_OH_2_)_2_PbI_4_ at 350 K, taken from ab initio molecular dynamics simulations at the end of 5 ps.


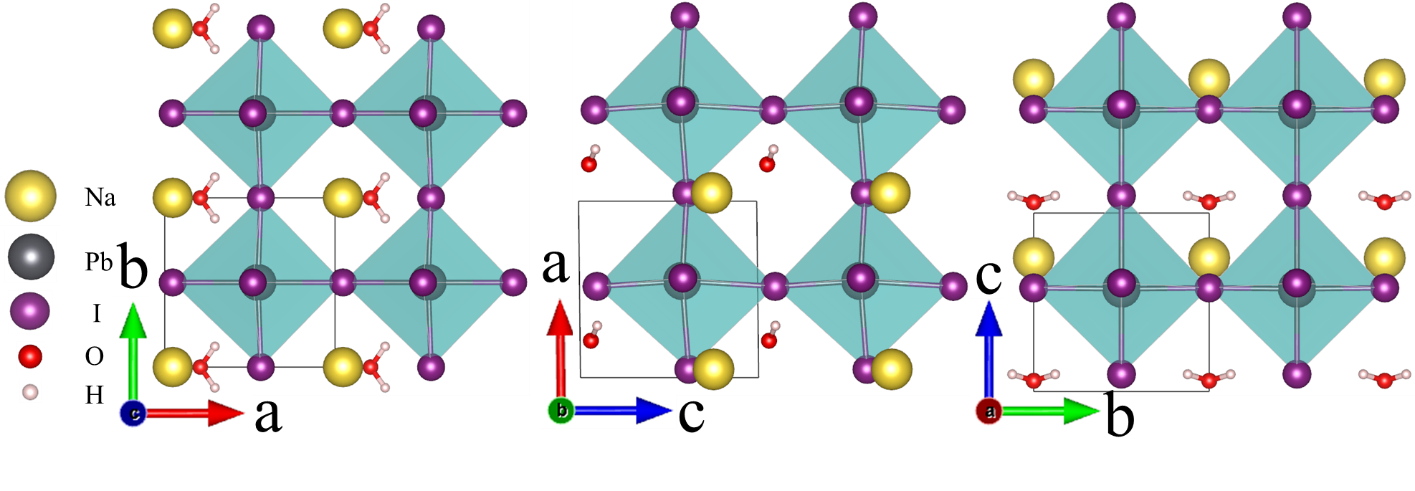


Figure S2. Geometric structure of Na(H_2_O)PbI_3_.

| (H_5_O_2_)PbI_3_ | (H_5_O_2_)SnI_3_ | (H_5_O_2_)PbBr_3_ | (C_2_H_7_O)PbI_3_ | (C_2_H_7_O)SnI_3_ | (C_2_H_7_O)PbBr_3_ | (CH_4_SH)PbI_3_ |
| --- | --- | --- | --- | --- | --- | --- |
| 1.00 | 1.02 | 1.03 | 0.97 | 0.99 | 0.99 | 0.95 |

Table S1. Tolerance factors of various cubic HOIPs.


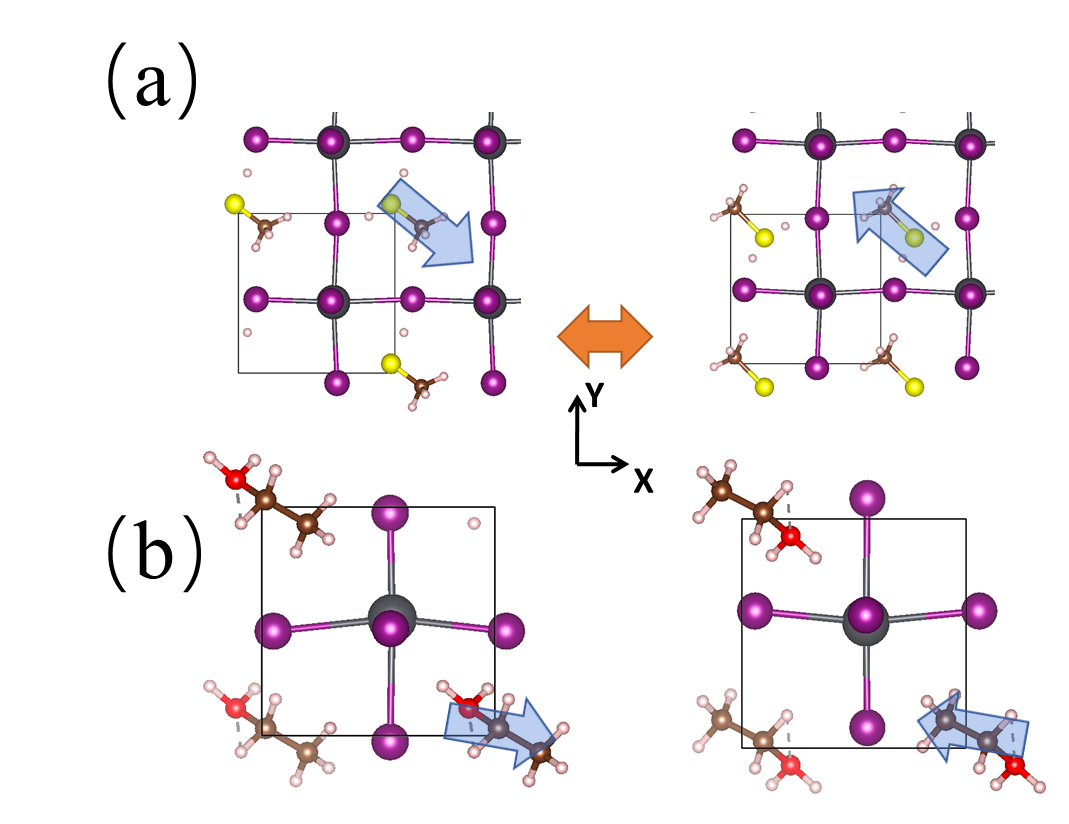


Figure S3. Ferroelectric switching of (a) (C_2_H_5_OH_2_)PbI_3_ and (b) (CH_4_SH)PbI_3_. The blue arrows denote the polarization directions.
